# The association between perceived cause of glaucoma and illness perceptions

**DOI:** 10.3389/fmed.2024.1363732

**Published:** 2024-04-04

**Authors:** Eunice Choe, Shervonne Poleon, Tracy Thomas, Lyne Racette

**Affiliations:** Department of Ophthalmology and Visual Sciences, Heersink School of Medicine, University of Alabama at Birmingham, Birmingham, AL, United States

**Keywords:** primary open-angle glaucoma, POAG, cause of disease, illness perception, BIPQ

## Abstract

**Purpose:**

The perceived cause of disease is an important factor that has been linked with treatment outcomes but has not been fully assessed in primary open-angle glaucoma (POAG). This study assessed the accuracy of patients’ perceived cause of POAG and identified associations between accuracy, illness perceptions, medication adherence, and quality of life (QoL).

**Methods:**

The Brief Illness Perception Questionnaire (BIPQ) was used to assess illness perceptions and asked patients to rank the three most important causes of their disease in order of importance. POAG risk factors recognized by the American Academy of Ophthalmology were used to code responses as accurate or inaccurate based on the following three methods: (1) coding any reported cause, regardless of rank, (2) coding only the first-ranked cause, and (3) coding and weighting all reported causes. Medication adherence was measured electronically. QoL was measured using the Glaucoma Quality of Life questionnaire. Mann–Whitney U test was used to detect differences in illness perceptions, medication adherence, and QoL between accuracy groups.

**Results:**

A total of 97 patients identified a cause of their POAG and were included in this analysis. A higher proportion of patients reported an accurate cause (86.6% using method 1, 78.4% using method 2, and 79.4% using method 3; all *p* < 0.001). Mean medication adherence was 86.0% ± 17.8 and was similar across accuracy groups (all *p* > 0.05). Using method 2 (*p* = 0.045) and method 3 (*p* = 0.028), patients who reported an accurate cause of their POAG believed that their illness would last for a longer time compared to patients who reported an inaccurate cause. Method 3 also revealed that patients who reported an accurate cause of their POAG had lower perceived understanding of their illness (*p* = 0.048) compared to patients who reported an inaccurate cause. There were no differences in QoL between accuracy groups (all *p* > 0.05).

**Conclusion:**

This study highlights the association between perceived cause of POAG and illness perceptions related to knowledge level and POAG duration. Future studies should assess associations between perceived cause of disease and other critical dimensions of illness perception.

## Introduction

Primary open-angle glaucoma (POAG) is the leading cause of irreversible blindness worldwide (1). POAG is a progressive neuropathy in which the optic nerve is damaged by chronically elevated pressure inside the eye (2). There is no cure for POAG. Apart from the subset of POAG patients without elevated eye pressure (normal tension glaucoma) (3), the disease is primarily managed through hypotensive eye drops that reduce pressure-induced damage (4). While ocular hypotensive therapy has been shown to delay POAG progression (5), consistent use of eye drops is a challenge for many patients. Medication adherence, which describes the degree to which patients use their eye drops as prescribed, is profoundly affected by factors such as high treatment cost (6) and complex treatment regimens (7). Research also indicates that illness perceptions may affect medication adherence (8), highlighting their important role in POAG management.

Illness perceptions have been extensively studied in chronic diseases such as diabetes and hypertension (8–12). A meta-analysis across 188 studies exploring illness perceptions revealed that accurate perceptions about the cause of disease were associated with reduced risk of hospitalization while perceptions about inability to control the course of a disease were associated with depression (11, 13, 14). However, in POAG, neither perceived cause of disease nor its relationship with other illness perceptions have been fully explored. In this study, we aimed to assess the accuracy of patients’ perceived cause of POAG and to identify associations between accuracy of perceived cause of POAG, illness perceptions, medication adherence, and quality of life (QoL).

## Methods

### Study participants

Patients were selected from a NIH-funded longitudinal study (EY025756) conducted at the University of Alabama at Birmingham (UAB) that aimed to improve the time-to-detection of POAG progression. This study—known hereafter as the parent study—was approved by the UAB Institutional Review Board, complied with HIPAA regulations, and adhered to the tenets of the Declaration of Helsinki. Eligibility criteria included: a clinical diagnosis of POAG, visual acuity better than 20/40 on the Snellen eye chart, mean deviation better than −12 dB, spherical and cylindrical refraction within 5D and 3D, respectively, and age > 18 years at baseline. To be included in the present study, patients in the parent study also needed to complete the Brief Illness Perception Questionnaire (BIPQ) (15), the Glaucoma Quality of Life-15 questionnaire (GQL-15) (16), and provide at least one response to the 9th BIPQ question, which assesses patients’ perceived cause of POAG. Patients with a history of secondary glaucoma, diseases affecting the visual field, intraocular surgery, or cognitive impairment were excluded from the parent study and from this analysis.

### Accuracy of perceived cause of POAG

Patients’ perceived cause of POAG was assessed using the BIPQ, which was self-administered. Each BIPQ question assessed one illness perception. The 9th question asked patients to rank the three most important causes of their illness. The following responses were encountered and excluded as patients did not indicate a cause for their POAG: “N/A,” “not sure,” “no idea,” “I have no clue,” “DK,” “DN,” and “I do not know.” A similar approach was used previously (13). The following POAG risk factors recognized by the American Academy of Ophthalmology (AAO) (17) were used to code responses as accurate or inaccurate: elevated intraocular pressure, older age, Black race, central corneal thickness, family history of POAG, myopia, and associated disorders (e.g., diabetes, hypertension, low ocular perfusion pressure, retinal vein occlusion). Two researchers (EC, LR) independently coded responses using three methods: 1) coding any response, regardless of rank, 2) coding only the first-ranked response, and 3) coding and weighting all responses. For method 3, accurate first-ranked responses were weighted as 3/3 points, accurate second-ranked responses as 2/3 points, accurate third ranked responses as 1/3 points, and inaccurate responses as 0/3 points. Weights, which were developed to give more importance to the higher-ranked responses, were averaged to yield a composite score out of 1. A threshold of 0.5 was used to establish accuracy. For example, if a patient provided three responses, with an accurate cause in ranks 1 and 2, but an inaccurate cause in rank 3, three points were allotted for the response in rank 1, 2 points were allotted for the response in rank 2, and 0 points were allotted for the response in rank 3. The composite score of 0.83 (5/6) was obtained and the patient was considered to have provided an accurate cause. This weighting was developed as a means to give more importance to responses provided in the first rank because patients were asked to report the three main causes for their glaucoma in order of perceived importance. Researchers compared coding results, and disagreements were resolved through adjudication by a third reviewer (SP). Coding was performed in Microsoft Excel (Redmond, WA, United States).

### Illness perceptions

**Figure 1:**
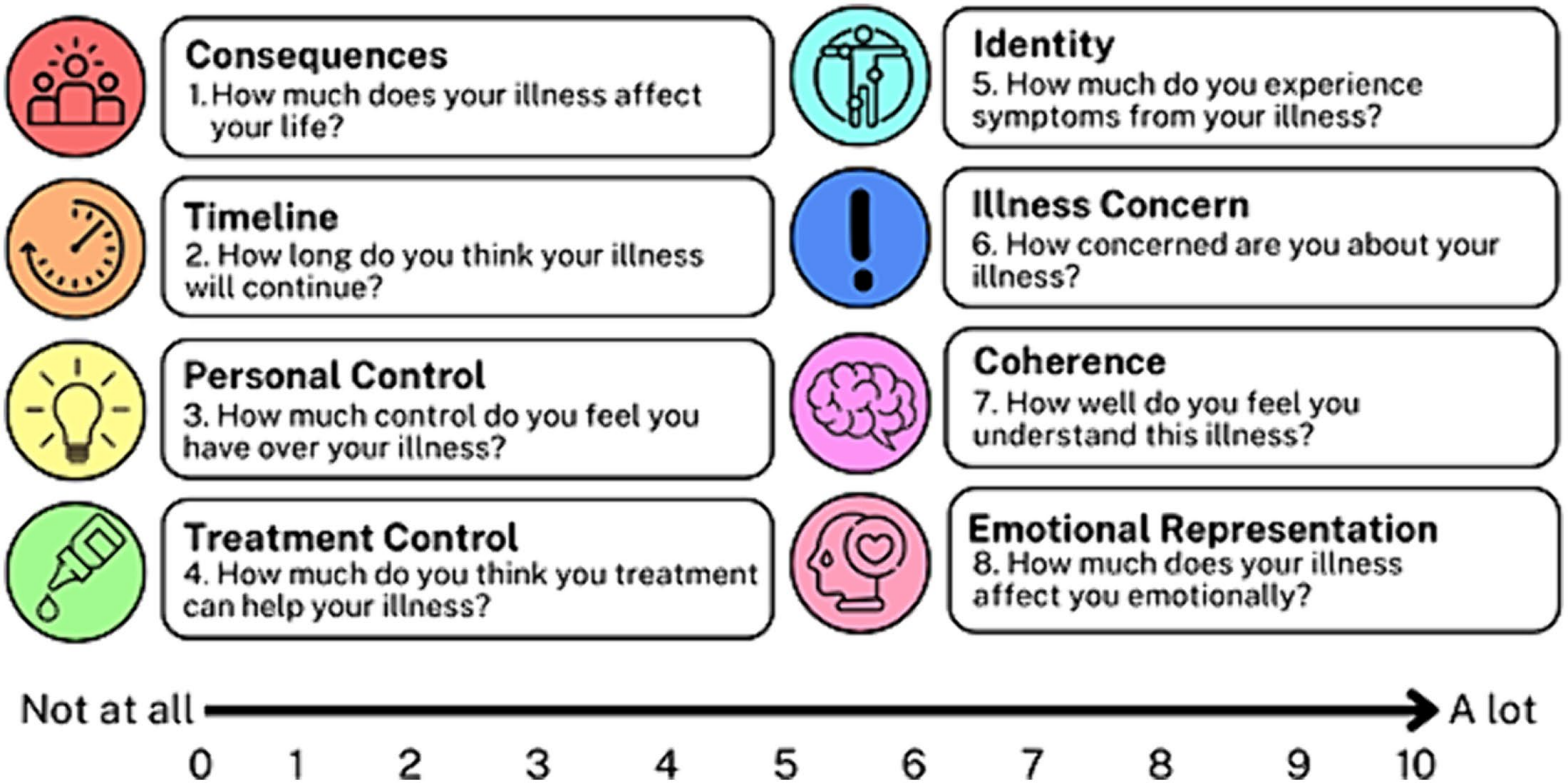
BIPQ illness perceptions 1–8. BIPQ, Brief illness perception questionnaire.

The BIPQ assessed the following eight perceptions: consequences, timeline, personal control, treatment control, identity, concern, understanding, and emotional response (Figure 1). Patients used a Likert scale ranging from 0 to 10 to indicate the extent of each perception. Questions 3, 4, and 7 were reverse-coded (had an opposite scale direction to the other questions) and were reviewed to detect response sets. Questions 3, 4, and 7 were then recoded per questionnaire guidelines. All Likert scores were summed to yield a total BIPQ score ranging from 0 to 80.

### Medication adherence

Medication adherence during the implementation phase was measured using Medication Event Monitoring System (MEMS) caps (Aardex; Liège, Belgium). The implementation phase describes the period between the moment patients fill their first prescription and the moment patients stop using their medication (18). Each patient was given one MEMS bottle per prescribed eye drop medication and was instructed to store their eye drop medication inside the MEMS bottle. The MEMS cap logged each opening of the MEMS bottle and this measurement served as a proxy for an instilled eye drop. Although imperfect, this method is considered to be the most objective (19). For each eye drop medication, daily medication adherence was calculated using the formula: $\frac{\mathrm{number}\;\mathrm{of}\;\mathrm{doses}\;\mathrm{taken}\;}{\mathrm{number}\;\mathrm{of}\;\mathrm{doses}\;\mathrm{prescribed}\;}\;\times \;100\%$ . Extra doses were excluded from the calculations. Daily medication adherence was then averaged over the total number of eye drops to calculate mean daily medication adherence for 14 months. The first 2 months of data (collected prior to the administration of the BIPQ) were excluded to minimize the Hawthorne effect, which describes research participants’ alteration of their behavior due to their awareness of being observed (20).

### Quality of life

The impact of POAG on patients’ QoL was assessed using the GQL-15, which spans four domains of visual disability: central and near vision, peripheral vision, dark adaptation and glare, and outdoor mobility. The study coordinator administered the GQL-15 and patients responded using a Likert scale ranging from 1 to 5. Per questionnaire guidelines, Likert responses were recoded using a numerical interval scale ranging from 0 (no difficulty) to 100 (severe difficulty) and then averaged to yield GQL-15 subscale scores. The GQL-15 total score was computed as the sum of the un-recoded Likert responses (21).

### Statistical analysis

Chi-squared test was used to determine whether there was a significant difference in the proportion of patients who reported an accurate vs. an inaccurate cause of their POAG. Mann–Whitney U test was used to detect difference in illness perceptions, medication adherence, or QoL among patients who reported an accurate vs. an inaccurate cause of POAG. All analyses were performed in SPSS version 29 (SPSS Inc.; Chicago, IL, United States). Alpha was set at 0.05.

## Results

Out of 114 patients, 97 were included in this analysis (17 patients only reported causes such as “I do not know” and were excluded from the analysis). A total of 31 patients provided three responses to the 9th BIPQ question, compared to 24 patients who provided two responses and 42 patients who provided only one response. Mean ± SD age was 69.1 ± 8.2 years and mean medication adherence was 86.0% ± 17.8. Table 1 presents patients’ baseline characteristics. Approximately 61% were female and approximately 52% were White. Sixty-six percent of patients attained a college degree or higher and approximately 36% reported a household income of $60,000 or more. Mean BIPQ total score was 29.4 ± 11.2 [maximum = 80]. Higher BIPQ total scores indicate a more daunting outlook on POAG. Mean GQL-15 total score was 24.3 ± 9.6 [54]. Higher GQL-15 total scores indicate a lower quality of life due to POAG.

**Table 1:** Patient characteristics.

Characteristics	Distribution
**Sample size, N (%)**	97 (100.0)
**Mean (SD) age, years**	69.1 ± 8.2
**Gender, N (%)**	
Female	59 (60.8)
Male	38 (39.2)
**Race, N (%)**	
White	50 (51.5)
Black	46 (47.4)
Other	1 (1.0)
**Education, N (%)**	
Less than high school	1 (1.0)
High school or GED	10 (10.3)
Trade/technical/vocational training	7 (7.2)
Some college	15 (15.5)
College degree (Associate and/or Bachelor’s)	42 (43.3)
Graduate/Professional degree	22 (22.7)
**Employment, N (%)**	
Retired	58 (59.8)
Employed Part-time	19 (19.6)
Employed Full-time	15 (15.5)
Not employed/unable to work	5 (5.2)
**Marital status, N (%)**	
Married	56 (57.7)
Not married/separated/divorced	41 (42.3)
**Household income, N (%)**	
Less than $10, 000	6 (6.2)
Less than $60,000	35 (36.1)
$60,000–$99,000	23 (23.7)
$100,000–149,00	6 (6.2)
More than $150,000	6 (6.2)
Not reported	27 (27.8)
**Questionnaires**	Mean (SD), median [IQR]
Total BIPQ score (*n* = 97)	29.4 (11.2), 28.0 [13.0]
GQL-15 score (*n* = 88)	24.3 (9.6), 20.5 [11.3]
Medication adherence (*n* = 62)	86.0 (17.8), 94.4 [18.7]

BIPQ, brief illness perception questionnaire; GED, general educational development; GQL-15, Glaucoma quality of life questionnaire-15; IQR, interquartile range; SD, standard deviation.

**Figure 2:**
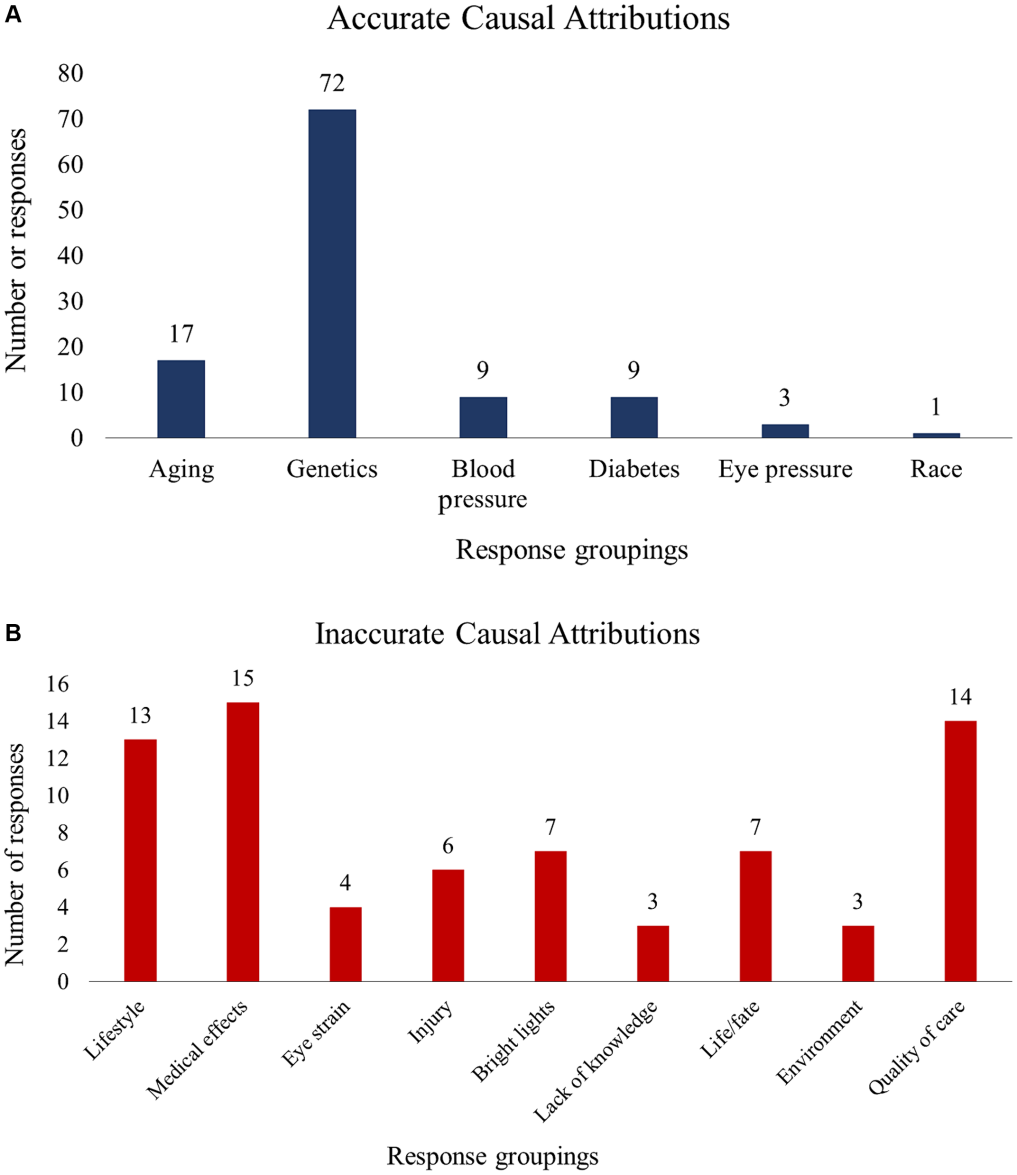
Summary of accurate **(A)** and inaccurate **(B)** causal disease attributions.

Using method 1, 84 patients (86.6%) reported an accurate cause of their POAG, compared to 76 patients (78.4%) using method 2 and 77 patients (79.4%) using method 3. Regardless of the method used, a larger proportion of patients reported an accurate vs. an inaccurate cause of their POAG (χ^2^ statistics were 51.97, 31.19, and 33.49 for methods 1, 2, and 3, respectively; all *p* < 0.001). Using method 1, we found that patients who reported an accurate cause of their POAG were younger (68.4 ± 8.2 years vs. 73.8 ± 6.1, *p* = 0.01). There were no age differences across accuracy groups when methods 2 or 3 were used. No significant differences were identified for QoL or any of the demographic variables. Figure 2 presents a summary of the accurate (A) and inaccurate (B) causal attributions. Accurate attributions included genetics/hereditary (*n* = 72), old age (*n* = 17), and diabetes (*n* = 9). Inaccurate attributions included lifestyle (*n* = 13), medical impacts/side effects (*n* = 15), and Quality of care (*n* = 14).

Table 2 presents the BIPQ illness perception scores. There were no differences between accuracy groups using method 1. Method 2 revealed that patients who identified an accurate cause of their POAG had higher scores for the Timeline perception, which reflects patients’ perceptions about how long their illness will last (8.9 ± 2.4 vs. 7.9 ± 3.1, *p* = 0.045). Method 3 revealed higher scores for the Timeline perception (9.0 ± 2.4 vs. 7.8 ± 3.1, *p* = 0.028) and lower scores for the Coherence perception (1.5 ± 2.1 vs. 1.9 ± 1.3, *p* = 0.048) in patients who reported an accurate vs. an inaccurate cause of their POAG. The coherence perception measures patients’ perceived level of knowledge about their illness.

**Table 2:** BIPQ subscale scores and accuracy of perceived POAG causality.

BIPQ perception	Accurate causality		Inaccurate causality		*p*-value
	Mean	Standard deviation	Mean	Standard deviation	
**Method 1**					
Consequence	2.0	2.4	1.8	2.4	0.692
Timeline	8.9	2.6	7.6	3.3	0.298
Personal control	3.8	3.1	2.3	2.5	0.187
Treatment control	1.6	2.1	1.4	1.7	0.929
Identity	2.3	2.8	1.9	2.3	0.694
Concern	7.2	3.1	6.9	3.5	0.978
Coherence	1.6	2.0	1.8	1.4	0.273
Emotional representation	2.6	2.9	0.8	0.8	0.189
Total score	30.1	11.3	24.5	8.6	0.266
**Method 2**					
Consequence	1.8	2.3	2.4	2.8	0.289
*Timeline*	*8.9*	*2.4*	*7.9*	*3.1*	*0.045*
Personal control	3.6	3.2	3.6	3.0	0.979
Treatment control	1.6	2.3	1.4	1.5	0.627
Identity	2.2	2.8	2.6	3.0	0.521
Concern	7.2	3.1	7.0	3.2	0.665
Coherence	1.6	2.1	1.8	1.4	0.099
Emotional representation	2.6	3.0	1.9	2.7	0.244
Total score	29.6	11.1	28.5	11.5	0.746
**Method 3**					
Consequence	1.8	2.3	2.4	2.8	0.392
*Timeline*	*9.0*	*2.4*	*7.8*	*3.1*	*0.028*
Personal control	3.7	3.2	3.5	3.0	0.798
Treatment control	1.6	2.3	1.5	1.5	0.658
Identity	2.2	2.8	2.8	3.0	0.363
Concern	7.3	3.1	6.9	3.2	0.473
*Coherence*	*1.5*	*2.1*	*1.9*	*1.3*	*0.048*
Emotional representation	2.6	3.0	1.9	2.8	0.195
Total score	29.7	11.1	28.4	11.8	0.649

Method (1) assessing any of the three responses, regardless of rank, (2) assessing only the first-ranked response, and (3) assessing all responses and weighting them based on rank. Italicized values indicate significant associations. Timeline represents patients’ perceived duration of their illness; Coherence represents patients’ perceived knowledge of their illness.BIPQ, brief illness perception questionnaire; POAG, primary open-angle glaucoma.

## Discussion

The majority of patients in this study reported an accurate cause of their POAG, such as “aging,” “genetics,” and “race.” This finding suggests that most patients may be well-informed about the cause of their disease, perhaps due to POAG-specific education provided by clinicians during clinic visits or sought out by patients themselves. The most common causal attribution was genetics/hereditary factors, which is encouraging as it may translate into more frequent eye examinations among persons with a family history of POAG and their relatives. However, as many as 20% of patients attributed their POAG to inaccurate causes such as “karma,” “eating habits” and “depression.” While not recognized as risk factors for POAG, responses such as “eating habits” may be references to vascular diseases, for example diabetes, which is associated with POAG (22–25). Other responses such as “karma” and “depression” speak to a less nuanced understanding of POAG.

In this study, the accuracy of patients’ perceived cause of POAG was determined based on clinical standards established by the American Academy of Ophthalmology (AAO). Therefore, we did not acknowledge the significance of other risk factors, including vitamin D3 levels (26–28), sleep apnea (29), exercise (30), and smoking (31). As a result, responses which may have alluded to these factors (“lifestyle” [n = 2], “diet” [n = 6], “injury” [n = 8], neglect of health [n = 11]) were coded as incorrect. This potential discrepancy highlights the complex nature of POAG and the need for continued research to fully elucidate the factors that play a role in POAG development and/or progression. Similarly, the connection between diabetes and POAG remains unclear, calling the accuracy of patients’ perceived link between them into question. Notwithstanding, diabetes is recognized as a potential risk factor for POAG by clinical bodies such as the AAO and the European Glaucoma Society (32). As such, providers may discuss diabetes with patients during clinic visits, leading to patients becoming more knowledgeable about the potential connection between the two conditions.

Our finding that patients who reported an accurate cause of their POAG were younger is intuitive, as older patients tend to have lower health literacy (33), and experience more literacy barriers such as declining vision and hearing (34). Therefore, interventions for improving health knowledge and health literacy in older patients with POAG may be beneficial as they are at greater risk for disease progression (35). We also found that patients who reported an accurate cause of their POAG believed that their disease would last longer compared to patients who reported an inaccurate cause. This finding is consistent with the lifelong nature of POAG (2) and suggests that patients who are informed about the cause of their disease may also be more informed about its other aspects. Clinicians may be able to successfully ascertain patients’ disease knowledge by assessing their causal attributions. Our analysis also revealed that patients who reported an accurate cause of their POAG had lower perceived knowledge about their disease compared to patients who reported an inaccurate cause. While this finding appears to be counterintuitive, it is possible that greater awareness of the cause of POAG reflects patients’ understanding of its complex nature, resulting in less confidence in their overall knowledge level. Overall, our findings suggest that disease causality may serve as a proxy for overall disease knowledge.

To the best of our knowledge, this is the first study in POAG to analyze patients’ perceived cause of POAG and its relationship to other clinical and behavioral variables. As such, this study is an important contribution to the limited data available on perceived disease causality and its relationship to illness perceptions in POAG. Another strength of this study is that electronic monitoring was used to measure medication adherence, which is believed to be the most objective method available (25). A final strength is that as there are no standardized methods for assessing accuracy of the perceived cause of disease, we developed three different methods, which helped to minimize bias. This study is not without limitations however, which include that the BIPQ was self-administered. Despite the presence of the study coordinator who carefully described the instructions, it is possible that some patients may not have fully comprehended them, particularly for the disease causality question. Another limitation is that we did not perform Bonferroni correction. However, our finding that a larger proportion of patients reported an accurate cause of their POAG is robust and would stand even after correcting for multiple comparisons. Other significant results would likely not remain significant, but we presented these findings without correction because this is an initial assessment of causal attribution in POAG, and the results may be useful in informing future research. A final potential limitation is that not all patients provided three responses to the causal question, perhaps indicating a rushed or superficial approach to completing the questionnaire, which may have biased our findings. It is possible however, that patients who only provided one response were certain that their POAG was caused by the one factor they reported.

Despite these limitations, this study highlighted the close association between perceived cause of POAG and other illness perceptions such as knowledge level and disease duration. We found that most patients identified an accurate cause of their POAG and that an accurate cause of POAG was associated with younger age, higher perceived duration of POAG, and lower perceived understanding of POAG. Future studies should aim to assess associations between perceived cause of disease and other critical dimensions of illness perception, and to determine how these may be associated with clinical outcomes other than medication adherence, such as disease severity and disease progression.

## Data availability statement

The de-identified data supporting the conclusions of this article will be made available by the authors, without undue reservation.

## Ethics statement

The studies involving humans were approved by the Institutional Review Board at the University of Alabama at Birmingham. The studies were conducted in accordance with the local legislation and institutional requirements. The participants provided their written informed consent to participate in this study.

## Author contributions

EC: Investigation, Methodology, Writing – original draft, Writing – review & editing, Formal analysis, Data curation. SP: Conceptualization, Data curation, Formal analysis, Investigation, Methodology, Project administration, Supervision, Writing – original draft, Writing – review & editing. TT: Data curation, Investigation, Writing – review & editing. LR: Conceptualization, Data curation, Formal analysis, Funding acquisition, Investigation, Methodology, Project administration, Supervision, Writing – original draft, Writing – review & editing.
